# Supplementary material for: fMRI insights into differential brain activation, executive function, and physical activity in older adults
Source: PLoS One. 2025 Jun 25;20(6):e0327163. doi: 10.1371/journal.pone.0327163 (PMC12194046; doi:10.1371/journal.pone.0327163)
Supplement: S1 File — (PDF) [file pone.0327163.s001.pdf]

ID	Group	Number	no	Gender	Age	Height	Weight	Education
24	1	2	102	2	65	165.00	86.00	9
26	1	4	104	1	60	176.00	80.00	9
27	1	5	105	2	63	156.00	65.00	9
28	1	6	106	1	63	170.00	80.00	12
29	1	7	107	1	61	175.00	80.00	12
30	1	8	108	1	63	170.00	75.00	12
31	1	9	109	2	60	160.00	66.00	9
33	1	11	111	1	67	175.00	85.00	12
35	1	13	113	2	63	160.00	60.00	12
36	1	14	114	2	64	162.00	60.00	12
37	1	15	115	1	69	168.00	85.00	6
38	1	16	116	1	59	178.00	82.50	12
39	1	17	117	1	64	173.00	75.00	12
40	1	18	118	2	64	158.00	53.00	12
41	1	19	119	1	64	172.00	75.00	15
43	1	21	121	1	62	175.00	80.00	12
44	1	22	123	2	63	161.00	60.00	12
46	1	24	202	1	63	168.00	79.00	12
47	1	25	401	2	66	172.00	65.00	9
48	1	26	402	2	63	160.00	68.00	12
1	2	1	301	1	20	180.00	55.00	15
2	2	2	302	1	24	173.00	70.00	16
3	2	3	303	1	23	173.00	63.00	16
5	2	5	305	2	27	156.00	46.00	19
6	2	6	306	1	27	170.00	75.00	19
7	2	7	307	2	22	164.00	65.00	16
8	2	8	308	2	20	160.00	50.00	16
9	2	9	309	2	22	163.00	52.00	16
10	2	10	310	2	23	174.00	58.00	16
11	2	11	311	1	25	178.00	80.00	19
12	2	12	312	2	21	169.00	60.00	16
13	2	13	313	1	24	163.00	54.00	18
14	2	14	314	1	23	186.00	90.00	16
15	2	15	315	2	23	175.00	65.00	16
16	2	16	316	2	23	168.00	80.00	16
17	2	17	317	1	19	178.00	65.00	13
18	2	18	318	1	23	175.00	75.00	17
19	2	19	319	1	25	175.00	61.00	16
20	2	20	320	2	21	174.00	75.00	16
21	2	21	321	1	25	180.00	70.00	16
22	2	22	322	1	23	175.00	67.00	19

fMRI insights into differential brain activation, executive function, and physical activity in older adults

IPAQ1	HighScore	ModerateS	WalkingScc	TotalScore	IPAQ2	MoCAtotal	MoCAExec	MoCAFluer
3	0.00	5880.00	5544.00	11424.00	2	26	0	2
3	1440.00	1440.00	1386.00	4266.00	2	22	0	1
3	840.00	4800.00	0.00	5640.00	2	26	1	1
2	0.00	0.00	1386.00	1386.00	1	26	1	2
3	0.00	3360.00	2425.50	5785.50	2	27	0	1
3	0.00	7560.00	693.00	8253.00	2	20	0	1
2	0.00	576.00	2079.00	2655.00	1	24	1	2
3	1980.00	840.00	1386.00	4206.00	2	21	1	1
3	0.00	0.00	4851.00	4851.00	2	24	0	1
2	0.00	0.00	2772.00	2772.00	1	22	1	1
3	0.00	2520.00	5544.00	8064.00	2	20	0	1
3	0.00	3600.00	693.00	4293.00	2	28	1	2
3	3360.00	3360.00	693.00	7413.00	2	23	1	1
2	0.00	0.00	2079.00	2079.00	1	25	1	1
2	0.00	2520.00	237.60	2757.60	1	27	1	2
3	19200.00	0.00	3465.00	22665.00	2	21	0	1
2	1440.00	0.00	1386.00	2826.00	1	24	0	2
3	23040.00	480.00	9702.00	33222.00	2	23	1	1
1	0.00	0.00	594.00	594.00	1	29	1	2
2	0.00	0.00	1386.00	1386.00	1	27	0	1
3	0.00	0.00	4158.00	4158.00	2	30	1	2
2	0.00	0.00	1188.00	1188.00	1	30	1	2
3	6720.00	1680.00	1386.00	9786.00	2	26	1	2
3	4032.00	672.00	3326.40	8030.40	2	28	1	2
2	0.00	0.00	2772.00	2772.00	1	28	1	2
2	480.00	0.00	1386.00	1866.00	1	28	1	2
3	0.00	360.00	4158.00	4518.00	2	28	1	2
3	1440.00	3600.00	5544.00	10584.00	2	26	1	2
3	384.00	3360.00	#NULL!	3744.00	2	28	1	2
3	1920.00	0.00	1386.00	3306.00	2	30	1	2
2	0.00	0.00	1386.00	1386.00	1	29	1	2
2	0.00	840.00	1386.00	2226.00	1	26	1	2
3	0.00	1344.00	10395.00	11739.00	2	28	1	2
3	5040.00	3360.00	792.00	9192.00	2	26	1	2
3	4320.00	0.00	1782.00	6102.00	2	27	1	2
3	0.00	720.00	2772.00	3492.00	2	29	1	2
2	0.00	0.00	2079.00	2079.00	1	28	1	2
2	840.00	504.00	693.00	2037.00	1	29	1	2
2	0.00	0.00	1386.00	1386.00	1	29	1	2
2	0.00	0.00	2772.00	2772.00	1	30	1	2
1	0.00	96.00	178.20	274.20	1	27	1	2

fMRI insights into differential brain activation, executive function, and physical activity in older adults

MoCAOrier	MoCACalcu	MoCAAbstı	MoCADela	MoCAVisuc	MoCANam	MoCAAtter	RBANStota	Immediate
6	3	3	2	3	4	3	205	45
6	3	2	2	2	3	3	200	40
6	3	2	4	3	4	2	201	39
6	3	3	2	2	4	3	178	38
6	3	1	3	6	4	3	179	26
5	3	2	1	2	3	3	159	25
6	2	2	2	2	4	3	171	32
5	2	3	4	0	3	2	179	31
5	3	3	2	3	4	3	199	22
5	3	1	2	3	3	3	145	27
6	3	0	2	2	4	2	148	24
5	3	3	4	3	4	3	189	30
4	3	3	3	2	4	2	187	36
6	3	3	3	1	4	3	211	40
6	3	3	3	3	4	2	209	36
6	3	3	1	0	4	3	154	32
5	3	2	3	2	4	3	160	29
6	3	2	3	1	3	3	167	35
6	3	3	5	2	4	3	209	41
6	3	3	4	3	4	3	227	43
6	3	3	5	3	4	3	231	47
6	3	3	5	3	4	3	259	52
6	3	3	1	3	4	3	228	46
6	3	3	3	3	4	3	228	53
6	3	3	3	3	4	3	228	48
6	3	3	4	3	3	3	275	57
6	3	3	3	3	4	3	261	52
6	3	3	2	3	3	3	262	55
6	3	3	3	3	4	3	244	52
6	3	3	5	3	4	3	254	44
6	3	3	4	3	4	3	251	51
5	3	3	2	3	4	3	237	50
6	3	3	5	3	4	1	261	46
5	3	3	2	3	4	3	266	54
5	3	3	3	3	4	3	271	54
6	3	3	4	3	4	3	266	52
6	3	3	3	3	4	3	252	49
6	3	3	4	3	4	3	235	43
6	3	3	4	3	4	3	289	50
6	3	3	5	3	4	3	215	47
6	3	3	2	3	4	3	249	50

fMRI insights into differential brain activation, executive function, and physical activity in older adults

visuospatia	Language	Attention	DelayedMe	FlankerCon	FlankerInco	FlankerCon	FlankerInco	back0CR
36	27	51	46	1.00	1.00	611.08	672.16	0.97
38	29	47	46	0.98	0.96	590.67	735.15	0.94
31	28	53	50	1.00	0.98	541.56	663.02	1.00
26	34	36	44	1.00	0.92	639.92	737.07	1.00
35	30	45	43	1.00	1.00	568.90	625.92	0.94
30	22	39	43	1.00	1.00	739.36	834.48	0.94
31	26	46	36	0.98	0.78	848.69	920.59	0.94
36	33	32	47	0.72	0.84	711.44	726.26	0.88
37	32	67	41	0.94	0.98	693.81	714.98	0.85
22	22	36	38	0.90	0.44	833.49	1017.18	0.85
31	26	27	40	0.96	1.00	668.27	723.12	0.97
29	36	45	49	1.00	1.00	577.36	640.80	1.00
37	23	44	47	1.00	0.98	577.64	652.57	1.00
34	35	56	46	1.00	1.00	550.98	626.18	1.00
33	34	56	50	1.00	1.00	603.68	730.54	0.94
27	23	32	40	0.98	0.94	627.82	700.32	0.97
34	25	35	37	0.98	0.26	683.59	1090.77	1.00
26	29	33	44	1.00	1.00	620.24	675.50	1.00
39	33	47	49	0.98	0.92	659.35	735.48	0.97
35	30	62	57	0.92	0.90	534.09	632.09	0.78
35	35	63	51	1.00	0.98	539.56	663.88	1.00
39	44	63	61	1.00	1.00	524.16	658.04	1.00
36	33	63	50	1.00	1.00	543.28	631.12	0.97
31	33	63	48	1.00	1.00	607.62	645.16	1.00
36	32	66	46	1.00	1.00	526.78	673.70	1.00
39	35	83	61	1.00	0.96	437.36	541.21	1.00
32	34	85	58	1.00	1.00	557.40	636.96	1.00
36	33	80	58	1.00	1.00	566.62	635.62	1.00
32	36	69	55	0.98	0.98	654.39	710.73	0.94
38	30	86	56	1.00	0.98	532.90	598.06	1.00
39	31	71	59	1.00	0.98	488.22	564.06	0.91
36	32	62	57	0.94	0.90	570.26	661.47	0.91
36	33	92	54	1.00	1.00	589.22	634.48	0.97
38	38	77	59	1.00	1.00	455.94	522.66	0.94
40	36	81	60	1.00	1.00	587.60	656.90	0.97
40	35	83	56	0.92	0.94	562.37	607.94	1.00
39	39	69	56	1.00	1.00	544.14	586.48	1.00
39	28	71	54	1.00	1.00	569.30	640.64	0.97
40	38	104	57	1.00	0.96	427.00	539.42	0.97
31	33	58	46	0.92	0.88	629.07	799.16	1.00
39	32	71	57	1.00	0.98	570.92	635.45	1.00

fMRI insights into differential brain activation, executive function, and physical activity in older adults

back1CR	back2CR	back0RT	back1RT	back2RT	shapeCR	colorCR	switchCR	shapeRT
0.91	0.67	801.94	927.36	924.40	0.94	0.78	0.69	1159.83
1.00	0.92	611.01	790.50	822.39	0.86	0.98	0.76	1071.33
0.94	0.92	607.56	688.34	721.24	0.98	1.00	0.79	1021.93
0.78	0.96	584.94	572.64	717.46	0.90	0.90	0.84	1195.90
0.88	0.79	858.81	952.69	959.93	0.82	0.80	0.86	1144.69
0.56	0.42	738.58	1107.63	1296.13	0.88	0.92	0.69	1255.88
0.84	0.75	724.40	833.12	902.76	0.90	1.00	0.80	1146.34
0.88	0.92	716.00	777.42	761.48	0.87	0.97	0.90	973.69
0.28	0.67	720.73	844.22	825.33	0.54	0.92	0.82	1431.75
0.56	0.63	860.82	816.63	757.40	0.80	1.00	0.92	1144.40
0.47	0.38	861.64	832.84	887.50	0.73	0.90	0.73	1062.95
0.88	0.88	551.66	661.35	808.37	0.76	0.94	0.69	1223.11
0.88	0.83	655.72	734.86	749.49	0.73	0.84	0.86	1198.53
0.72	0.71	659.91	841.23	870.03	1.00	1.00	0.82	964.03
0.91	0.96	603.67	686.54	712.38	0.96	1.00	0.88	983.79
0.53	0.71	822.04	981.68	885.94	0.42	0.90	0.69	1386.75
0.78	0.54	812.35	862.95	860.92	0.67	0.92	0.61	1209.60
0.84	0.92	636.63	731.20	754.44	0.82	0.88	0.82	1243.01
0.94	0.75	604.42	662.06	813.92	0.94	1.00	0.98	1188.14
0.84	0.88	646.64	632.56	861.05	0.94	0.90	0.84	1022.88
0.81	0.46	572.44	654.19	530.63	0.98	1.00	0.96	841.52
1.00	0.92	635.57	633.64	724.58	0.98	1.00	1.00	925.62
0.84	0.79	697.47	808.36	911.67	1.00	0.78	0.94	1221.51
1.00	0.79	644.22	746.14	1236.13	1.00	1.00	0.94	909.53
0.94	0.96	570.19	630.95	612.92	1.00	1.00	0.96	1011.48
0.94	0.96	532.97	558.77	447.19	0.96	0.88	0.92	797.56
1.00	0.92	513.88	610.93	723.97	1.00	1.00	0.94	792.84
0.94	0.75	685.35	717.56	777.57	0.94	0.90	0.92	1000.93
1.00	0.83	687.02	745.00	964.83	1.00	0.98	1.00	949.44
0.94	0.92	518.29	646.52	856.11	0.90	0.92	1.00	904.98
0.94	0.88	572.70	578.25	805.46	0.96	0.90	0.96	865.76
0.78	0.83	622.38	611.58	533.84	0.90	0.96	0.86	830.34
1.00	0.96	698.23	731.00	697.78	1.00	1.00	0.92	970.71
0.88	0.79	482.38	545.65	738.61	0.88	0.90	0.88	693.05
1.00	0.79	649.13	722.57	738.89	0.98	1.00	1.00	1008.80
1.00	0.92	613.44	644.43	658.50	0.92	0.94	0.98	798.39
1.00	0.96	615.44	634.57	716.88	1.00	0.94	0.98	872.48
1.00	0.96	650.33	742.61	710.95	1.00	1.00	0.98	1010.04
1.00	0.96	530.86	615.25	649.95	0.96	0.94	0.96	723.37
1.00	0.46	607.60	724.54	1059.06	0.98	0.90	0.84	1040.27
0.88	0.96	610.32	625.90	605.32	1.00	0.94	0.96	863.90

fMRI insights into differential brain activation, executive function, and physical activity in older adults

colorRT	switchRT
1116.65	1310.50
1135.45	1274.08
930.46	1080.94
1045.86	1196.78
1063.82	1244.51
1287.45	1402.25
1011.80	1361.41
1005.76	1233.90
1178.42	1399.86
1230.57	1446.07
1041.66	1355.39
957.71	1120.52
1129.71	1169.08
1024.00	1126.90
905.40	1031.96
1215.07	1204.24
1183.83	1394.19
1036.82	1167.01
959.28	1215.46
923.81	1149.77
888.34	843.59
854.75	989.96
1184.17	1338.27
976.15	1104.43
991.71	1068.32
806.21	876.74
798.32	977.54
997.68	1074.56
871.95	988.38
886.89	938.92
796.56	931.39
942.44	1075.39
1014.53	1037.15
760.80	784.34
910.80	1066.23
764.37	807.00
862.24	948.51
1129.94	1231.81
738.46	829.14
1017.42	1170.59
948.57	1010.39

fMRI insights into differential brain activation, executive function, and physical activity in older adults
