# Supplementary material for: fMRI insights into differential brain activation, executive function, and physical activity in older adults
Source: PLoS One. 2025 Jun 25;20(6):e0327163. doi: 10.1371/journal.pone.0327163 (PMC12194046; doi:10.1371/journal.pone.0327163)
Supplement: Attachment — Submitted filename: PONE-D-24-57669_Review.pdf [file pone.0327163.s002.pdf]

Title: fMRI insights into differential brain activation and physical activity in older adults

Author: Huiqi SONG, Jie FENG, Yingying WANG, Qichen ZHOU, Chenglin ZHOU, Jing JIN

Date of review: 02/21/2025

Strengths of the article

1. Relevance of the subject

The article deals with a subject of major interest in cognitive neuroscience and ageing, namely the impact of physical activity on executive function and brain activation. The research question is well motivated and supported by references.

2. Well-detailed methodology

The study uses well-established tasks to assess executive function (Flanker, N-back, Switching). The use of fMRI to analyse brain activation is relevant and justified.

3. Interesting results

The study shows interesting differences in brain activation depending on the level of physical activity. The finding that physically active adults require less activation to perform executive tasks is consistent with the hypothesis of better cognitive efficiency.

Weaknesses and suggestions for improvement

1. General comments regarding the document

Q1. There are several spelling errors: the term executive function**s** is plural. It is also written "recreation" instead of "reaction" in several places in the document.

2. Title page

"fMRI insights into differential brain activation and physical activity in older adults"

Q2. Why don't you mention the term "executive functions" in the long title?

3. Abstract

"Methods: This cross-sectional study involved 51 Han Chinese adults (22 young, 31 older) who were categorized as physically active (≥3000 MET-min/week) or inactive (<3000 METmin/ week)."

Q3. Please explicit the term "MET-min/week".

4. Methods
- Study design and participants

“The inclusion criteria were: (1) Han Chinese adults aged 18 years or older; (2) had more than 10 years of education”

Q4. What was the exact upper age limit in the young group? What were the exact lower and upper age limits in the older group?

Q5. The mean educational level in the older group is 11.10 +/- 1.97. It means that 1 or more participant(s) had less than 10 years of education. How do you explain that discrepancy?

“A total of 51 participants were included, including 22 young adults and 31 older adults.”

Q6. I do not understand this sentence as 22+31 = 53.

Q7. How was the sample size calculated? This information must be indicated according to the Strengthening the Reporting of Observational Studies in Epidemiology (STROBE) statement.

- Imaging data acquisition and analysis

Q8. Why did you not include imaging in the “Measurements” section?

Q9. What corrections for multiple testing were applied? They must be reported in each table.

Q10. How did you select ROIs?

- Statistical methods

Q11. Statistical methods must be described (see the STROBE statement). There is no indication of which statistical tests were used to analyze data other than brain imaging data. Nor is it indicated which dispersion measure is presented in the article: standard deviation or standard error of the mean? I recommend to add a section 2.4. “Statistical methods” or “Statistical analyses”.

5. Results

“Among 52 participants who agreed to participate in this study”

Q12. Why were 51 participants included and 52 participants who agreed to participate?

Q13. What was the mean physical activity level in each group? This must be indicated in the Table 1.

Q14. How did you choose the physical activity cut-off between physically active and physically inactive (3000 MET-min/week)? Is it relevant to choose to use the same cut-off for young and older groups? One may argue that an older person will be considered as active with less activity than a younger person.

Q15. Results must indicate the statistics value (for example: t value if a Student t test has been used or the F value if an ANOVA has been performed) wherever necessary. P values are not sufficient.

6. Table 1.

"Data are presented as mean (SD) or number (percentage)."

Q16. I do not understand this sentence. It seems that some lines indicate means +/- SD and that other lines indicate the number of participants with percentages in brackets. Is it correct?
