# Supplementary material for: fMRI insights into differential brain activation, executive function, and physical activity in older adults
Source: PLoS One. 2025 Jun 25;20(6):e0327163. doi: 10.1371/journal.pone.0327163 (PMC12194046; doi:10.1371/journal.pone.0327163)
Supplement: Attachment — Submitted filename: PEER REVIEW REPORT (PONE-D-24-57669).pdf [file pone.0327163.s003.pdf]

# PEER REVIEW REPORT

**Manuscript Title:** fMRI Insights into Differential Brain Activation and Physical Activity in Older Adults
**Manuscript Number:** PONE-D-24-57669

## Revision Suggestion

### Justification for the 3000 MET-min/week Threshold

The 3000 MET-min/week threshold used to classify physical activity levels is not explicitly justified and lacks supporting scientific references.

**Revision Suggestion:**

The rationale for this threshold should be supported by previous studies or recommendations from health organizations (e.g., WHO, ACSM).

Does this threshold have a specific significance for cognitive health or brain function? If prior research has demonstrated positive effects of a certain MET level on brain function, this should be clearly stated.

### Conclusion

The study is well-structured and addresses an important topic; however, minor revisions are required to improve the justification for the 3000 MET-min/week threshold.

**Decision:** Minor Revisions Required.
