# Supplementary material for: fMRI insights into differential brain activation, executive function, and physical activity in older adults
Source: PLoS One. 2025 Jun 25;20(6):e0327163. doi: 10.1371/journal.pone.0327163 (PMC12194046; doi:10.1371/journal.pone.0327163)
Supplement: Attachment — Submitted filename: PEER REVIEW REPORT-2- (PONE-D-24-57669).pdf [file pone.0327163.s005.pdf]

PEER REVIEW REPORT

Manuscript Title: fMRI Insights into Differential Brain Activation and Physical Activity in Older Adults

Manuscript Number: PONE-D-24-57669

Revision Suggestion

Clarification and Justification for the 3000 MET-min/week Threshold
The authors have provided references to support their use of the 3000 MET-min/week threshold (Andersen et al., 2021; Macfarlane et al., 2007). While these sources confirm the general applicability of the IPAQ-SF and its validity in certain populations, they do not sufficiently explain why this particular cutoff was chosen in the context of cognitive health or brain activation.

This threshold is not commonly used in neuroimaging or cognitive neuroscience research. Therefore, its selection in this study should be either:

- clearly justified with reference to prior work or guidelines related to brain health (e.g., WHO, ACSM), or

- explicitly presented as a novel, hypothesis-driven approach to examine possible neurocognitive associations.

Providing this clarification will strengthen the conceptual alignment between the study's aims and its methodological framework.

Conclusion
The study is well-structured and addresses an important research question. However, minor revisions are necessary to improve the justification of the 3000 MET-min/week threshold and to ensure stronger methodological transparency.

Decision: Minor Revisions Required.
