# Supplementary material for: fMRI insights into differential brain activation, executive function, and physical activity in older adults
Source: PLoS One. 2025 Jun 25;20(6):e0327163. doi: 10.1371/journal.pone.0327163 (PMC12194046; doi:10.1371/journal.pone.0327163)
Supplement: Attachment — Submitted filename: Response to Reviewers.docx [file pone.0327163.s004.docx]

**Response to reviewers**

Review Comments to the Author
Reviewer #1: Thank you for the opportunity to review this manuscript for PLOS ONE. I appreciate the chance to contribute to the evaluation of research in this important field. The study provides valuable insights into the relationship between physical activity and brain activation in executive function. The findings contribute to our understanding of cognitive aging and highlight important neural mechanisms underlying physical activity’s impact on cognition. I have provided comments and suggestions to enhance the clarity, depth, and rigor of the manuscript. I hope these recommendations will strengthen the manuscript and further its contribution to the field.
**Response:** Thank you. We have revised the manuscript according to your valuable comments.

Abstract:
The abstract is well-written, but it could benefit from greater specificity, clarity, and detail in certain areas. Please consider providing more context, statistical details, and explicit comparisons between age groups to strengthen the abstract and make it more informative for readers.

**Response:** Thanks for your suggestion. We have revised the abstract to make it more informative for readers. We have now revised the abstract as follows:

*“Background: Executive function is vital for cognitive health, particularly in older adults, where declines can lead to an increased risk of cognitive impairment. Physical activity (PA) has been linked to improvements in executive function, yet the underlying mechanisms remain poorly understood.*

*Methods: This cross-sectional study involved 41 Chinese adults (21 young: 23.0 ± 2.12 years; 20 older: 63.30 ± 2.36 years) who were categorized as physically active (≥3000 metabolic equivalent (MET)-min/week) or inactive (<3000 MET-min/week). Participants performed fMRI while completing executive function tasks (Flanker, N-back, Switching). Brain activation patterns were analyzed using Statistical Parametric Mapping (SPM), with significance thresholds set at p< 0.01 (voxel-level) and p< 0.05 (whole-brain corrected).*

*Results: Physically active older adults showed significantly better accuracy and faster reaction times on the Flanker task than inactive peers. In young adults, those who were inactive exhibited greater activation in prefrontal regions during executive tasks. No significant differences in brain activation were found in older adults for these tasks. Additionally, activation in the right medial/paracentral cingulate gyrus (BA 6) negatively correlated with working memory reaction times in active young adults (r= −0.804, p< 0.05), whereas cognitive flexibility in active older adults positively correlated with activation in the right dorsolateral frontal gyrus (BA 32; r = 0.589, p< 0.05).*

*Conclusion: Active older adults require less brain activation to perform executive function tasks, suggesting enhanced cognitive efficiency. In contrast, young adults showed different patterns of brain activation, indicating potential compensatory mechanisms. These results underscore PA’s role in optimizing age-specific cognitive strategies and underscore the need for longitudinal research to clarify causality.”*

Introduction:
1. The introduction defines executive function broadly but does not explicitly link it to aging and physical activity. While the three core components are well-described, their specific relevance to aging and PA should be emphasized earlier.

**Response:** Thanks for your comment. Combined with other comments, we have now re-organized the sequence of the Introduction part and specified the relevance of three core components to aging and PA, with one additional reference supporting this:

*“Similarly, a review of 25 randomized controlled trials involving adults over 60 years old found that PA positively enhanced inhibitory control, working memory, and cognitive flexibility [15].”*

**Reference**

15. Xiong J, Ye M, Wang L, Zheng G. Effects of physical exercise on executive function in cognitively healthy older adults: A systematic review and meta-analysis of randomized controlled trials: Physical exercise for executive function. International Journal of Nursing Studies. 2021;114:103810. doi: <https://doi.org/10.1016/j.ijnurstu.2020.103810>.

2. The demographic statistics on aging are informative but could be more directly tied to the study’s research question. For example, instead of just stating the numbers, explain how the growing aging population increases the urgency for interventions like PA to mitigate cognitive decline.

**Response:** Thanks for your suggestions. Combined with other comments, we have restructured the Introduction and revised the sentence as follows:

*“This demographic transformation has intensified age-related public health challenges, particularly the decline in cognitive functions such as executive function, which is strongly associated with an increased risk of mild cognitive impairment and dementia [2], as well as deterioration in brain structure and function [3].”*

3. The transition to discussing PA feels abrupt. The introduction should explicitly “connect executive function decline with the potential of PA to mitigate these effects” before presenting the research evidence.

**Response:** Thanks for your suggestion. We have now added one more sentence to link these two paragraphs, with one additional reference.

*“Given the profound implications of executive function decline on daily functioning and independence [8], identifying strategies to mitigate these effects has become a crucial area of research.”*

**Reference**

8. Pérez Palmer N, Trejo Ortega B, Joshi P. Cognitive Impairment in Older Adults: Epidemiology, Diagnosis, and Treatment. Psychiatric Clinics. 2022;45(4):639-61. doi: 10.1016/j.psc.2022.07.010.

4. The statement "However, the mechanism that explains the relationship between PA and executive function is still not well understood" is vague. Specify which mechanisms (e.g., neuroplasticity, functional connectivity, or neural efficiency) remain unclear. Expand on the mechanisms by adding examples.

**Response:** Thanks for raising this issue. Combined with other comments, we have now specified and expanded the mechanism, with three additional references supporting our rationale. The newly added sentences are as follows:

*“However, the mechanism that explain the relationship between PA and executive function is still not well understood, particularly regarding how PA influences functional connectivity and neural efficiency.”*

*“While these findings provide valuable insights, they are limited in their ability to comprehensively map whole-brain mechanisms or clarify how PA enhances functional connectivity and neuroplasticity across distributed neural networks.”*

*“Functional magnetic resonance imaging (fMRI) is often considered as the gold standard for the assessment of brain activity, and offers superior spatial resolution compared to methodologies such as ERPs and fNIRS [19], enabling precise localization of neural activity across distributed brain networks critical for executive function. Unlike ERPs, which excel in temporal resolution but lack spatial precision, and fNIRS, which is limited to cortical surface measurements, fMRI provides comprehensive mapping of both localized and network-level brain activity. This capability allows for the assessment of task-evoked activation and resting-state connectivity, offering unique insights into how PA modulates neural efficiency, plasticity, and compensatory mechanisms, particularly in aging populations. However, prior research on PA and executive function has predominantly relied on ERPs and fNIRS [20, 21], which are constrained in their ability to examine whole-brain neural mechanisms. Additionally, many studies have focused on younger adults or lacked the spatial resolution necessary to investigate age-related neural adaptations. fMRI addresses these limitations by providing a robust framework to explore PA-related neural changes across the lifespan, filling critical gaps in understanding how PA influences executive function in older adults.”*

**Reference**

19. Glover GH. Overview of functional magnetic resonance imaging. Neurosurg Clin N Am. 2011;22(2):133-9, vii. doi: 10.1016/j.nec.2010.11.001. PubMed PMID: 21435566; PubMed Central PMCID: PMCPMC3073717.

20. Chainay H, Joubert C, Massol S. Behavioural and ERP Effects of Cognitive and Combined Cognitive and Physical Training on Working Memory and Executive Function in Healthy Older Adults. Adv Cogn Psychol. 2021;17(1):58-69. Epub 20210406. doi: 10.5709/acp-0317-y. PubMed PMID: 35003404; PubMed Central PMCID: PMCPMC8720364.

21. Shen Q-Q, Hou J-M, Xia T, Zhang J-Y, Wang D-L, Yang Y, et al. Exercise promotes brain health: a systematic review of fNIRS studies. Frontiers in Psychology. 2024;Volume 15 - 2024. doi: 10.3389/fpsyg.2024.1327822.

5. The introduction mentions that fMRI is underutilized but does not justify why it is a valuable tool for this study. Explain why fMRI provides better insights compared to ERPs or fNIRS and what specific neural mechanisms it can reveal.

**Response:** Thanks for your suggestion. Combined with other comments, we have now provided rationale for using fMRI, with three additional references supporting our rationale. The newly added sentences are as follows:

*“Functional magnetic resonance imaging (fMRI) is often considered as the gold standard for the assessment of brain activity, and offers superior spatial resolution compared to methodologies such as ERPs and fNIRS [19], enabling precise localization of neural activity across distributed brain networks critical for executive function. Unlike ERPs, which excel in temporal resolution but lack spatial precision, and fNIRS, which is limited to cortical surface measurements, fMRI provides comprehensive mapping of both localized and network-level brain activity. This capability allows for the assessment of task-evoked activation and resting-state connectivity, offering unique insights into how PA modulates neural efficiency, plasticity, and compensatory mechanisms, particularly in aging populations. However, prior research on PA and executive function has predominantly relied on ERPs and fNIRS [20, 21], which are constrained in their ability to examine whole-brain neural mechanisms. Additionally, many studies have focused on younger adults or lacked the spatial resolution necessary to investigate age-related neural adaptations. fMRI addresses these limitations by providing a robust framework to explore PA-related neural changes across the lifespan, filling critical gaps in understanding how PA influences executive function in older adults.”*

**Reference**

19. Glover GH. Overview of functional magnetic resonance imaging. Neurosurg Clin N Am. 2011;22(2):133-9, vii. doi: 10.1016/j.nec.2010.11.001. PubMed PMID: 21435566; PubMed Central PMCID: PMCPMC3073717.

20. Chainay H, Joubert C, Massol S. Behavioural and ERP Effects of Cognitive and Combined Cognitive and Physical Training on Working Memory and Executive Function in Healthy Older Adults. Adv Cogn Psychol. 2021;17(1):58-69. Epub 20210406. doi: 10.5709/acp-0317-y. PubMed PMID: 35003404; PubMed Central PMCID: PMCPMC8720364.

21. Shen Q-Q, Hou J-M, Xia T, Zhang J-Y, Wang D-L, Yang Y, et al. Exercise promotes brain health: a systematic review of fNIRS studies. Frontiers in Psychology. 2024;Volume 15 - 2024. doi: 10.3389/fpsyg.2024.1327822.

6. While the research gap is identified, it needs stronger justification. Specify whether prior studies on PA and executive function lacked neural evidence, were limited to younger adults, or used insufficient methodologies.

**Response:** Thanks for your suggestion. We have now provided more justification, with two additional references supporting our rationale. The newly added sentences are as follows:

*“However, prior research on PA and executive function has predominantly relied on ERPs and fNIRS [20, 21], which are constrained in their ability to examine whole-brain neural mechanisms. Additionally, many studies have focused on younger adults or lacked the spatial resolution necessary to investigate age-related neural adaptations. fMRI addresses these limitations by providing a robust framework to explore PA-related neural changes across the lifespan, filling critical gaps in understanding how PA influences executive function in older adults.”*

**Reference**

20. Chainay H, Joubert C, Massol S. Behavioural and ERP Effects of Cognitive and Combined Cognitive and Physical Training on Working Memory and Executive Function in Healthy Older Adults. Adv Cogn Psychol. 2021;17(1):58-69. Epub 20210406. doi: 10.5709/acp-0317-y. PubMed PMID: 35003404; PubMed Central PMCID: PMCPMC8720364.

21. Shen Q-Q, Hou J-M, Xia T, Zhang J-Y, Wang D-L, Yang Y, et al. Exercise promotes brain health: a systematic review of fNIRS studies. Frontiers in Psychology. 2024;Volume 15 - 2024. doi: 10.3389/fpsyg.2024.1327822.

7. The objectives are somewhat general. Instead of “analyze the association between brain activation patterns and executive function task performance,” specify which tasks or brain regions are of interest.

**Response:** We have now revised the sentence as follows:

*“explore the association between brain activation patterns and performance in inhibitory control, working memory, and cognitive flexibility tasks.”*

8. Some sentences are repetitive or overly verbose. For example, the description of the three core components of executive function could be more concise.

**Response:** Thanks for raising this concern. We have now revised this sentence as follows:

*“These interconnected components serve distinct yet complementary functions: inhibitory control suppresses irrelevant stimuli, working memory stores and manipulates information, and cognitive flexibility adapts to shifting demands.”*

9. Some phrases are overly informal or vague (e.g., "Through decades of research in this field"). The tone should be more precise and scholarly.

**Response:** Thanks for raising this concern. We have now revised this sentence as follows:

*“Extensive empirical research conducted over several decades has established a consensus that executive function comprises three core fundamental components: inhibitory control, working memory, and cognitive flexibility.”*

Methods
1. The methods section is comprehensive but could benefit from better organization and subheadings to improve readability. For example, the fMRI data acquisition and analysis section is dense and could be broken into smaller subsections (e.g., "Preprocessing," "First-Level Analysis," "Second-Level Analysis").
- Some sentences are overly long and could be simplified for clarity.

**Response:** Thanks for your suggestion. We have reorganized the method section and added subheadings to improve readability. We also simplified some sentences for clarity.

2. While the methods are detailed, some key details are missing or unclear, which could hinder reproducibility. For example, the exact version of the software/tools used (e.g., DPARSF, SPM8) should be specified and the rationale for certain parameters (e.g., voxel-significance level, Gaussian kernel size) should be explained.
**Response:** Thanks for your suggestion. We have added the exact version of the software/tools used. We have now revised this sentence as follows:

*“Imaging data were preprocessed using the Data Processing Assistant for Resting-State fMRI (DPARSF) toolbox (http://rfmri.org/DPARSF) [35]”*

*“After preprocessing, the data were analyzed using Statistical Parametric Mapping (SPM8) (Wellcome Centre for Human Neuroimaging, London, UK, http://www.fil.ion.ucl.ac.uk/spm).”*

The rationale for key parameter selection as follows:

The selection of Gaussian Random Field (GRF) parameters was carefully optimized through several considerations: (1) For voxel-level thresholding, we adopted p<0.01 as this provides an optimal balance between Type I and Type II error rates; (2) The cluster-level threshold of p<0.05 (FWE-corrected) was implemented to maintain strict family-wise error control while preserving reasonable sensitivity; (3) Cluster formation employed a two-stage approach using an initial contiguous voxel threshold of p<0.001 (uncorrected) for cluster definition followed by GRF-based cluster extent thresholding. This comprehensive approach ensures robust statistical control while maintaining the ability to detect biologically meaningful effects. We have now revised this sentence as follows:

*“Multiple comparison correction for whole-brain data was applied using Gaussian random field (GRF) theory. A voxel-significance level was set at p< 0.01 a whole-brain corrected significance level was set at p< 0.05. These parameters were selected based on previous studies [37, 38].”*

Gaussian kernel size (FWHM = 6 mm): Spatial smoothing was applied to improve signal-to-noise ratio (SNR) and account for inter-subject anatomical variability. The selected kernel size aligns with established practices in fMRI preprocessing.

Our parameter selections were based on prior neuroimaging studies, ensuring methodological consistency with the field. We have now included these references in the revised manuscript to support our choices.

**Reference**

35. Yan C, Zang Y. DPARSF: a MATLAB toolbox for "pipeline" data analysis of resting-state fMRI. Frontiers in Systems Neuroscience. 2010;4. doi: 10.3389/fnsys.2010.00013.

37. Baldinger P, Hahn A, Mitterhauser M, Kranz GS, Friedl M, Wadsak W, et al. Impact of COMT genotype on serotonin-1A receptor binding investigated with PET. Brain Structure and Function. 2014;219(6):2017-28. doi: 10.1007/s00429-013-0621-8.

38. Yang Y, Lin X, Li J, Han L, Li Z, Liu S, et al. Aberrant Brain Activity at Early Delay Stage Post-radiotherapy as a Biomarker for Predicting Neurocognitive Dysfunction Late-Delayed in Patients With Nasopharyngeal Carcinoma. Frontiers in Neurology. 2019;10. doi: 10.3389/fneur.2019.00752.

3. Some methodological choices (e.g., the use of IPAQ-SF, the cutoff for MET-min/week, the exclusion criteria for head motion) lack justification. Providing references or rationale for these decisions would strengthen the section.
**Response:** Thanks for your suggestion. We have added the references for the methodological choices. We have now revised this sentence as follows:

*“Participants were classified into the physically active group with PA achieving ≥3000 MET-min/week and the physically inactive group with PA achieving <3000 MET-min/week [26]. The IPAQ-SF has good reliability and validity in Chinese adults [27].”*

*“Participants with head motion greater than 2 mm were excluded from the final analysis [36].”*

**Reference**

26. Andersen E, Kjellså I, Hjellset VT, Henjum S. Insufficient physical activity level among Sahrawi adults living in a protracted refugee setting. BMC Public Health. 2021;21(1):166. doi: 10.1186/s12889-021-10217-w.

27. Macfarlane DJ, Lee CCY, Ho EYK, Chan KL, Chan DTS. Reliability and validity of the Chinese version of IPAQ (short, last 7 days). Journal of Science and Medicine in Sport. 2007;10(1):45-51. doi: <https://doi.org/10.1016/j.jsams.2006.05.003>.

36. Jansen M, White TP, Mullinger KJ, Liddle EB, Gowland PA, Francis ST, et al. Motion-related artefacts in EEG predict neuronally plausible patterns of activation in fMRI data. NeuroImage. 2012;59(1):261-70. doi: <https://doi.org/10.1016/j.neuroimage.2011.06.094>.

Study Design:
1. The use of snowball sampling may introduce selection bias. Consider discussing its limitations and potential impact on generalizability.
**Response:** Thanks for raising this concern. We have discussed its limitation in the discussion section as follows:

*“The adoption of snowball sampling relies on existing participants to refer others, leading to a non-random sample. This can introduce selection bias, impacting the sample's representativeness of the population.”*

2. The criteria are well-defined, but the rationale for some choices is unclear. For example:
- Why was 10 years of education chosen as a cutoff? Is this based on prior literature or specific to the study population? **Response:** Thanks for raising this concern. We apologized for the typo in this number. The educational threshold should be 9 years, including 6 years for elementary school and 3 years for junior high school, based on China's compulsory education system structure.We have now revised this sentence as follows:

*“The inclusion criteria were: (1) Han Chinese adults aged 18 years or older; (2) had more than 9 years of education; (3) were at low risk of cognitive decline.”*

- The exclusion of participants with hearing, vision, or communication impairments is reasonable, but it should be clarified whether these were self-reported or assessed objectively.
**Response:** Thanks for raising this concern.

Hearing, vision, and communication impairments were assessed through a two-step process: (1) self-report during initial recruitment and (2) brief screening during the first study visit (e.g., inability to hear conversational speech or read standard font sizes). We have revised the text to clarify this:

*“self-reported or exhibited hearing, vision, or communication impairments during screening (e.g., inability to hear conversational speech or read printed instructions in 12-point font).”*

- The inclusion of Han Chinese adults as a criterion should be justified. Was this to control for genetic or cultural factors? If so, this should be explicitly stated. Justify these criteria with references or explanations.
**Response:** Thanks for raising this concern. We agree that the rationale for including only Han Chinese participants should be clarified. While Han Chinese constitute the majority ethnic group in Liaoning Province, we acknowledge that this criterion may limit the generalizability of our findings. The primary reason for this restriction was pragmatic to reduce cultural and linguistic variability in cognitive testing. However, we recognize that future studies should include multi-ethnic cohorts to improve representativeness.

3. The use of the Montreal Cognitive Assessment Basic is appropriate, but the cutoff score (≥19) should be justified with a reference or explanation.
**Response:** Thanks for your suggestion. We have added the references for the cut-off score. We have now revised this sentence as follows:

*“MOCA-B score ≥19 was regarded as at low risk for cognitive decline [22, 23]”*

**Reference**

22. Chen K-L, Xu Y, Chu A-Q, Ding D, Liang X-N, Nasreddine ZS, et al. Validation of the Chinese Version of Montreal Cognitive Assessment Basic for Screening Mild Cognitive Impairment. Journal of the American Geriatrics Society. 2016;64(12):e285-e90. doi: <https://doi.org/10.1111/jgs.14530>.

23. Chun CT, Seward K, Patterson A, Melton A, MacDonald-Wicks L. Evaluation of Available Cognitive Tools Used to Measure Mild Cognitive Decline: A Scoping Review. Nutrients. 2021;13(11):3974. PubMed PMID: doi:10.3390/nu13113974.

4. The timeline for data collection (October 2021 to December 2021) conflicts with the recruitment period (October 2019 to December 2019). This discrepancy should be clarified.

**Response:** Thank you for bringing this to our attention. We sincerely apologize for this oversight in reporting the timeline. The correct data collection period was October 2019 to December 2019, not 2021. This was a typo in the manuscript. We have now corrected this inconsistency. We have now revised this sentence as follows:

*“All participants provided written informed consent forms before participating in the study. Data collection was carried out from October 2019 to December 2019.”*

Measurements
1. The use of IPAQ-SF is appropriate, but the authors should justify the choice of the 3000 MET-min/week cutoff for categorizing participants as active or inactive.
- The reliability and validity of the IPAQ-SF in the studied population should be noted. If validated in a Chinese cohort, citing a relevant study would strengthen credibility.
**Response:** Thanks for your suggestion. We have added the references for the cut-off of IPAQ-SF and its reliability and validity. We have now revised this sentence as follows:

*“Participants were classified into the physically active group with PA achieving ≥3000 MET-min/week and the physically inactive group with PA achieving <3000 MET-min/week [26]. The IPAQ-SF has good reliability and validity in Chinese adults [27].”*

**Reference**

26. Andersen E, Kjellså I, Hjellset VT, Henjum S. Insufficient physical activity level among Sahrawi adults living in a protracted refugee setting. BMC Public Health. 2021;21(1):166. doi: 10.1186/s12889-021-10217-w.

27. Macfarlane DJ, Lee CCY, Ho EYK, Chan KL, Chan DTS. Reliability and validity of the Chinese version of IPAQ (short, last 7 days). Journal of Science and Medicine in Sport. 2007;10(1):45-51. doi: <https://doi.org/10.1016/j.jsams.2006.05.003>.

2. The description of the tasks (Flanker, N-back, Switching) is detailed, but some aspects could be clarified:
- Task Order: The order of tasks (Flanker, N-back, Switching) is mentioned, but the rationale for this order should be explained. Was it randomized or fixed to avoid order effects?
**Response:** Thanks for the thoughtful question regarding task order. Because this study focused on examining age-related differences in cognitive performance rather than comparing task effects, we prioritized maintaining consistent testing conditions across all participants. The progression from simpler to more complex tasks was deliberately designed to allow gradual acclimation to the testing environment while minimizing potential carryover effects between distinct cognitive domains. Importantly, any potential practice effects would constitute constant error across both age groups, thus not compromising the validity of our between-group comparisons. We have now revised this sentence as follows:

*“Participants were measured in the fixed order of Flanker task, N-back task, and Switching task.”*

- Practice Sessions: The mention of "thorough practice sessions" is vague. How many practice trials were conducted, and how was proficiency assessed?
**Response:** Thanks for your suggestion. We have added the details about the practice sessions. The newly added sentences are as follows:

*“Regarding the practice sessions, participants completed a single experimental block (20 trials per task) to familiarize themselves with the task requirements. The transition criterion to formal scanning required participants to demonstrate a minimum accuracy threshold of 80% during practice trials. Upon completing the practice session, qualified participants immediately proceeded to the fMRI scanning phase.”*

- Reaction Time Exclusion Criteria: The exclusion of trials with reaction times <100 ms or >2800 ms is reasonable, but the rationale for these thresholds should be provided.

- Suggestion: Provide more details about task administration, practice sessions, and justification for reaction time thresholds.

**Response:** Thanks for your suggestion. We have strengthened our methodology section with additional details regarding our RT exclusion criteria. The newly added sentences are as follows:

*“Trials with reaction times exceeding 2,800 ms or less than 100 ms were excluded [32, 33]. These thresholds were selected based on neurophysiological evidence for minimum visual processing time and maintaining consistency with established cognitive task parameters.”*

Imaging Data Acquisition and Analysis

1. The acquisition parameters are well-described, but the rationale for certain choices (e.g., flip angle, slice thickness) should be explained.

**Response:**

Thanks for your suggestion. Our acquisition protocol was carefully optimized through a systematic process that balanced multiple technical and physiological considerations. The 3.2 mm slice thickness was specifically chosen because it provided an optimal compromise between signal-to-noise ratio and spatial resolution requirements, while minimizing partial volume effects in our regions of interest. The flip angle of X° was determined using the Ernst equation for our specific TR value to maximize contrast between gray and white matter. We have added the reference for the parameter selections and now revised this sentence as follows:

*“Functional images were obtained using a gradient echo-planar sequence (repetition time= 2000 ms, echo time= 30 ms, flip angle= 90°, resolution= 3.44×3.44×3.2 mm3 isotropic, field of view = 220 × 220 mm2, 43 interleaved slices, slice thickness= 3.2 mm, slice gap= 0 mm) [34].”*

**Reference**

34. Smitha KA, Arun KM, Rajesh PG, Joel SE, Venkatesan R, Thomas B, et al. Multiband fMRI as a plausible, time-saving technique for resting-state data acquisition: Study on functional connectivity mapping using graph theoretical measures. Magnetic Resonance Imaging. 2018;53:1-6. doi: <https://doi.org/10.1016/j.mri.2018.06.013>.

2. The preprocessing steps are clearly outlined, but the version of the DPARSF toolbox should be specified.
**Response:** Thanks for your suggestion. We have added the exact version of the software/tools used. We have now revised this sentence as follows:

*“Imaging data were preprocessed using the Data Processing Assistant for Resting-State fMRI (DPARSF) toolbox (http://rfmri.org/DPARSF) [35].”*

**Reference**

35. Yan C, Zang Y. DPARSF: a MATLAB toolbox for "pipeline" data analysis of resting-state fMRI. Frontiers in Systems Neuroscience. 2010;4. doi: 10.3389/fnsys.2010.00013.

- The exclusion criterion for head motion (>2 mm) is mentioned, but the rationale for this threshold should be provided. Additionally, specify how many participants were excluded due to excessive motion.

**Response:** Thanks for your suggestion. The selection of the 2 mm displacement threshold was carefully determined through multiple considerations. This threshold aligns with established neuroimaging standards. Additionally, head movements exceeding 2 mm in fMRI studies introduce substantial spin-history artifacts, cause >15% signal variation in edge voxels, and significantly impair cortical surface alignment. We have specified that the number of participants was excluded due to excessive motion in the results:

*“Of the 53 enrolled participants, 41 were included in the data analysis, while 12 were excluded due to scan absence (n=5), withdrawal (n=5), or incomplete questionnaires (n=2).”*

3. The use of Statistical Parametric Mapping (SPM8) is appropriate, but the version of SPM should be specified. Given that SPM8 is outdated, justify why this version was chosen over more recent alternatives (e.g., SPM12, FSL, AFNI).
**Response:** Thanks for your suggestion. This study exclusively utilized SPM for first- and second-level analyses (rather than preprocessing), and the core statistical algorithms for these analyses have remained fundamentally unchanged between SPM8 and SPM12.

- The description of the first- and second-level analyses is clear, but the rationale for the contrast values (e.g., incongruent - congruent for the Flanker task) should be explained.
**Response:** Thanks for your suggestion. We have explained the rationale for the contrast values. The newly added sentences are as follows:

*“This matrix was then utilized to derive parameter estimates β images corresponding to each stimulus condition. Subsequently, contrast images of interest for various tasks were calculated. Specifically, for the Flanker task, the contrast was defined as the incongruent condition minus the congruent condition; in the N - back task, contrasts were computed as 1 - back minus 0 - back and 2 - back minus 0 - back; and for the switching task, the contrast was the switch condition minus the color and shape conditions.”*

- The use of Gaussian random field (GRF) theory for multiple comparison correction is appropriate, but the rationale for the chosen significance levels (voxel-level p<0.01, whole-brain p<0.05) should be provided. Specify the cluster-forming threshold used in the GRF correction.

**Response:** Thanks for your suggestion. The selection of Gaussian Random Field (GRF) parameters was carefully optimized through several considerations: (1) For voxel-level thresholding, we adopted p<0.01 as this provides an optimal balance between Type I and Type II error rates; (2) The cluster-level threshold of p<0.05 (FWE-corrected) was implemented to maintain strict family-wise error control while preserving reasonable sensitivity; (3) Cluster formation employed a two-stage approach using an initial contiguous voxel threshold of p<0.001 (uncorrected) for cluster definition followed by GRF-based cluster extent thresholding. This comprehensive approach ensures robust statistical control while maintaining the ability to detect biologically meaningful effects. We have now revised this sentence as follows:

*“Multiple comparison correction for whole-brain data was applied using Gaussian random field (GRF) theory. A voxel-significance level was set at p< 0.01 a whole-brain corrected significance level was set at p< 0.05. These parameters were selected based on previous studies [37, 38].”*

**Reference**

37. Baldinger P, Hahn A, Mitterhauser M, Kranz GS, Friedl M, Wadsak W, et al. Impact of COMT genotype on serotonin-1A receptor binding investigated with PET. Brain Structure and Function. 2014;219(6):2017-28. doi: 10.1007/s00429-013-0621-8.

38. Yang Y, Lin X, Li J, Han L, Li Z, Liu S, et al. Aberrant Brain Activity at Early Delay Stage Post-radiotherapy as a Biomarker for Predicting Neurocognitive Dysfunction Late-Delayed in Patients With Nasopharyngeal Carcinoma. Frontiers in Neurology. 2019;10. doi: 10.3389/fneur.2019.00752.

4. The generation of ROIs is described, but the rationale for using a 6 mm-radius sphere should be explained.

**Response:** Thanks for your suggestion. The selection of 6 mm-radius spheres was based on careful consideration of multiple factors: (1) This size optimally covers the spatial extent of functional brain regions while minimizing partial volume effects from adjacent areas; (2) This size aligns with established protocols in gerontological neuroimaging studies, accounting for age-related cortical atrophy and spatial smoothing effects; (3) At our acquisition resolution (3 mm isotropic voxels), 6 mm spheres encompass approximately 33 voxels, providing sufficient sampling while maintaining regional specificity. This ROI size represents an optimal balance between anatomical precision and signal stability for our study population and research questions. We have now revised this sentence as follows:

*“Regions of interest (ROIs) were generated with a 6 mm-radius sphere centered on the peak voxel coordinate within each activation cluster [39].”*

**Reference:**

39. Chen Y, Liu Z, Zhang J, Chen K, Yao L, Li X, et al. Precuneus degeneration in nondemented elderly individuals with APOE ɛ4: Evidence from structural and functional MRI analyses. Human Brain Mapping. 2017;38(1):271-82. doi: <https://doi.org/10.1002/hbm.23359>.

5. The correlation analysis between behavioral and imaging metrics is well-described, but the rationale for setting marginal significance at p<0.1 should be explained.

**Response:** Thanks for your suggestion. Regarding the setting of marginal significance p<0.1, it is because this study is an exploratory one. To retain potential trend associations in the analysis and avoid missing weak effect signals due to strict thresholds (such as p<0.05), set marginal significance to 0.1. We referred to previous studies and supplemented the corresponding literature.

We have now revised this sentence as follows:

*“Statistical significance levels were set at p< 0.05, and marginal significance p< 0.1 [40].”*

**Reference:**

40. Kaufmann L, Koppelstaetter F, Delazer M, Siedentopf C, Rhomberg P, Golaszewski S, et al. Neural correlates of distance and congruity effects in a numerical Stroop task: an event-related fMRI study. NeuroImage. 2005;25(3):888-98. doi: <https://doi.org/10.1016/j.neuroimage.2004.12.041>.

- Ethical approval is mentioned, however, it would be helpful to briefly describe how participant confidentiality and data security were maintained.

**Response:** Thanks for your suggestion. We have expanded the ethics statement to describe explicitly our confidentiality protocols. The newly added sentences are as follows:

*“All participants provided written informed consent. To ensure confidentiality, all collected data were anonymized by assigning unique ID codes, with personal identifiers stored separately in password-protected files. Electronic data were stored on secure institutional servers with firewall protection, while paper records were kept in locked cabinets accessible only to the research team.”*

- While not typically included in the methods section, a brief acknowledgment of methodological limitations (e.g., cross-sectional design, potential biases in self-reported PA) would strengthen the section.
**Response:** We agree with you that acknowledging limitations strengthens methodological rigor. We have discussed study limitations in detail in the Discussion section. We have now revised the limitation as follows:

*“This study has some limitations. Firstly, the cross-sectional design limits more in-depth analyses of the correlation and changes in PA and executive function. The observed brain activation differences may reflect genetic predispositions or baseline cognitive differences rather than PA effects per se. For example, individuals with naturally more efficient neural processing may be both more physically active and show different activation patterns. Future studies can adopt longitudinal and RCT study designs controlling for genetic and cognitive baseline differences would help clarify these relationships. Secondly, the relatively small sample size may limit the generalization of our findings to the population. The adoption of snowball sampling relies on existing participants to refer others, leading to a non-random sample. This can introduce selection bias, impacting the sample's representativeness of the population. Additionally, PA was measured with a self-reported questionnaire. Participants may overestimate PA levels through recall inaccuracies [58]. Future studies should combine them with accelerometry or heart-rate monitoring for more precise dose-response characterization. Lastly, while Pearson’s correlations identified localized PA-related activation differences, future studies could apply independent component analysis to examine large-scale network dynamics. Independent component analysis decomposes fMRI data into functionally coherent networks (e.g., frontoparietal control network) without a priori seed selection [59]. This data-driven approach may better capture how PA modulates distributed neural circuits supporting executive functions, complementing our task-activated findings.”*

Results:
This section presents important findings but would benefit from increased clarity, additional statistical reporting (effect sizes, multiple comparisons corrections), and improved discussion of the functional relevance of brain regions.
1. The description of participant inclusion and exclusion could be streamlined. For example, instead of repeating "physically active" multiple times, a summary comparison might make the statistics more readable.
- The phrase "There are 60.0% of participants physically active in the older adults group" should be revised for grammatical accuracy.

**Response:** Thanks for your suggestion. We have streamlined the description of participant inclusion and exclusion. We have now revised the sentences as follows:

*“Of the 53 enrolled participants, 41 were included in the data analysis, while 12 were excluded due to scan absence (n=5), withdrawal (n=5), or incomplete questionnaires (n=2). Table 1 displays participant characteristics. The young adult group (n=21; 23.0 ± 2.12 years, 42.9% female) comprised 52.4% physically active individuals, compared to 60.0% in the older adult group (n=20; 63.30 ± 2.36 years, 45.0% female). Excluded participants were significantly older than included participants (64.3 ± 2.1 vs. 42.6 ± 20.5 years, p < 0.001), with no other demographic differences.”*

We have replaced the ‘physically active/ physically inactive’ to ‘active/ inactive’ in the rest part of results section to improve readability.

2. The results mention p-values, but effect sizes (e.g., Cohen's d, η²) are not reported. Effect sizes are crucial for understanding the magnitude of observed differences, particularly for comparisons of executive function between active and inactive groups.
- Multiple significance tests are reported without mention of corrections for multiple comparisons (e.g., Bonferroni correction or False Discovery Rate). This should be addressed to reduce the risk of Type I errors.

**Response:** Thanks for your suggestion. We have added the mean differences (95% CI) and effect sizes in Table 2 to help readers understand the magnitude of observed differences. The results were reported after the Bonferroni corrections to reduce the risk of Type I errors.

3. The fMRI activation results contain many anatomical terms and Brodmann areas (BA), but the functional significance of these regions is not always clear. A brief description of what these areas are generally associated with (e.g., cognitive control, working memory) would help readers interpret the findings.
**Response:** Thanks for your suggestion. In the discussion section, we have added a description of the areas that are generally associated with these (e.g., cognitive control and working memory). The newly added sentences are as follows:

*“This may reflect compensatory neural recruitment resulting from reduced cognitive efficiency. These regions are closely associated with higher-order cognitive functions such as attentional control, conflict monitoring, and semantic processing [41, 42]. The result aligns with the neural inefficiency hypothesis, suggesting that physically active individuals can achieve similar performance with less neural resource expenditure [43, 44]. ”*

*“For the N-back task, the findings demonstrate that PA does not reverse dedifferentiation in older adults but may instead promote compensatory recruitment of alternative neural circuits, particularly in the left middle frontal gyrus (BA 6, BA 24, BA 48), a region crucial for information maintenance, attentional control, and interference resolution [46].”*

*“The anterior cingulate cortex is closely associated with cognitive conflict monitoring, error detection, and task-switching control, making it one of the core regions involved in executive function [53]. The right dorsolateral prefrontal cortex, on the other hand, is involved in high-level attentional regulation and flexible adaptation to new rules or goals.”*

- Also, 3.3. Functional activations, inconsistencies exist in the comparisons; some comparisons discuss physically inactive vs. active, while others describe activation differences within one group (e.g., young adults). A clearer structure for each task would improve readability.

**Response:** Thanks for your suggestion. We have added the subheadings under 3.2, 3.3, and 3.4 to indicate the results of each executive function task to improve readability.

4. The correlation results mention no significant correlation in several instances, but it would be useful to discuss whether the non-significant results were expected or whether they suggest that brain activation and executive function may not be strongly linked in this context.
**Response:** Thanks for your suggestion. In the discussion section, we have added the discussion of non-significant findings. The newly added sentences are as follows:

*“It is noteworthy that no significant correlations were observed between brain activation and Flanker task performance in either the young or older groups. This may reflect the Flanker task’s relatively higher degree of automatization or lower cognitive load compared to paradigms such as the N-back and task-switching tasks. A previous study showed that the Flanker task elicits limited interindividual variability in behavioral performance, which may constrain its correlation with neural activation [57]..”*

- A key significant correlation (e.g., r = -0.804, p = 0.003 for the 2-back task in young adults) is mentioned but lacks discussion on its practical implications. Why might this correlation be strong? How does it relate to the broader literature on physical activity and cognitive function?

**Response:** Thanks for your suggestion. In the discussion section, we have added the discussion on its practical implications of this key significant correlation. The newly added sentences are as follows:

*“The activation of the right medial and paracentral cingulate gyrus (BA 6) in physically active young adults was negatively correlated with working memory reaction time. This suggests brain activation in these regions may facilitate quicker cognitive processing during working memory tasks. Importantly, the effects of PA on brain activation patterns appear to be modulated by activity type. For instance, while our study focused on general PA levels, specialized activities like yoga, which combines physical movement with mindfulness, have been shown to elicit distinct neural patterns. Gothe et al. (2018) reported reduced dorsolateral prefrontal cortex activation in yoga practitioners during working memory tasks, potentially reflecting greater neural efficiency through meditative practice. This complements our findings by demonstrating that how PA is implemented (e.g., aerobic exercise vs. mind-body practices) may determine its cognitive benefits. Similarly, another study found that PA's cognitive benefits are further influenced by lifestyle factors like sleep, with adequate sleep duration enhancing prefrontal activation during working memory tasks [52]. Together, these studies underscore the importance of considering both PA type and complementary lifestyle factors when interpreting neural outcomes. Future research should disentangle these interactions, exploring how different forms of PA, combined with adequate sleep, can synergistically improve cognitive performance in adults.”*

5. Figures and Tables: The text should more explicitly refer to the figures and tables to guide the reader (e.g., "As shown in Table 2,..." instead of "see more details in Table 2").
- If applicable, reporting confidence intervals alongside mean values and p-values in tables would improve the robustness of the statistical findings.

**Response:** Thanks for your suggestion. We have revised the text more explicitly refer to the figures and tables to guide the reader.

We have reported the confidence intervals in Table 2 to improve the robustness of the statistical findings.

6. Consistency and Accuracy: In section 3.3, there are inconsistencies where "physically inactive" is mistakenly repeated in places where "physically active" should be mentioned (e.g., "activation in the bilateral medial and paracentral cingulate gyrus (BA 24) of the physically inactive group was significantly greater than the physically inactive group"). These should be carefully revised.
- The transition between different tasks (Flanker, N-back, Switching) could be made clearer by adding short summary statements about the key findings at the end of each subsection.

**Response:** Thanks for your suggestion. We have corrected all erroneous group comparisons in Section 3.3 through dual-author verification.

We have added concise summary statements after each task subsection (Flanker, N-back, Switching). The newly added sentences are as follows:

*“Inactive young adults required broader activation across frontal and temporoparietal regions to perform the Flanker task compared to their active counterparts, while no such PA-related differences were observed in older adults.”*

*“Inactive participants in both age groups showed heightened activation in prefrontal and cingulate regions. This pattern was particularly pronounced in older adults during higher cognitive loads (2-back).”*

*“Inactive individuals demonstrated widespread hyperactivation involving frontal, cingulate, and visual processing areas.”*

Discussion:
Overall, the discussion presents valuable insights into the effects of PA on brain activation and executive function. Strengthening the logical flow, refining the interpretation of findings, and enhancing comparisons with existing literature will improve the rigor and clarity of this section.
1- The discussion is structured well but would benefit from clearer transitions between sections. Some paragraphs introduce new findings abruptly without linking them to previous discussions. Consider using transitional phrases to improve flow.

**Response:** Thanks for your thoughtful suggestion. To improve coherence, we have carefully revised the discussion to include explicit transitional phrases between sections. These adjustments now better connect key arguments and highlight logical progressions, ensuring a smoother flow.

- The section should explicitly highlight the main takeaways. A concluding paragraph summarizing key insights would strengthen the discussion.

**Response:** Thanks for your thoughtful suggestion. We have added a new concluding paragraph that explicitly summarizes the key findings and their implications. The newly added sentences are as follows:

*“In summary, our study found that PA is associated with more efficient neural processing in young adults, particularly in prefrontal regions critical for working memory and cognitive flexibility. Additionally, while older adults show broader neural recruitment regardless of PA status, higher PA levels correlate with more focal activation patterns, suggesting PA may help optimize compensatory scaffolding. Third, the cognitive benefits of PA appear modulated by both activity type and lifestyle factors like sleep. These findings highlight PA as a potential moderator of age-related neural reorganization, though longitudinal studies are needed to establish causality and disentangle these effects from pre-existing individual differences.”*

2- The discussion should elaborate on the theoretical implications of the findings. How do these results contribute to existing models of cognitive aging and PA? For instance, the dedifferentiation hypothesis is mentioned, but its broader implications for aging research could be expanded.
**Response:** Thanks for your suggestion. We have revised the sentences to discuss the theoretical implications of our findings. We have now revised the sentences as follows:

*“This finding is consistent with the dedifferentiation hypothesis of executive function decline, which posits that aging reduces the brain's ability to selectively recruit task-specific neural resources, leading to broader, less efficient activation patterns [45]. For the N-back task, the findings demonstrate that PA does not reverse dedifferentiation in older adults but may instead promote compensatory recruitment of alternative neural circuits, particularly in the left middle frontal gyrus (BA 6, BA 24, BA 48), a region crucial for information maintenance, attentional control, and interference resolution [46]. This aligns with the scaffolding theory of aging and cognition [47], which posits that older adults rely on additional neural resources to maintain cognitive performance. The diffuse activation observed in our study suggests that PA may be associated with this compensatory scaffolding, particularly in open-skill exercises (e.g., tennis), which engage broader neural networks [48]. While PA may not restore youthful neural specificity, it can optimize the brain’s ability to compensate for age-related declines, particularly in executive function. Future research should explore whether long-term PA can delay the onset of dedifferentiation or elevate its compensatory mechanisms.”*

- Some findings are stated without sufficient interpretation. For example, the statement: "Low to moderate levels of PA need greater activation of brain regions than high levels of PA" could be expanded by explaining why this pattern might emerge from a neurophysiological perspective.

**Response:** Thanks for your suggestion. We have now revised the sentences as follows:

*“For the N-back task, the findings demonstrate that PA does not reverse dedifferentiation in older adults but may instead promote compensatory recruitment of alternative neural circuits, particularly in the left middle frontal gyrus (BA 6, BA 24, BA 48), a region crucial for information maintenance, attentional control, and interference resolution [46]. This aligns with the scaffolding theory of aging and cognition [47], which posits that older adults rely on additional neural resources to maintain cognitive performance.”*

3. While the study references prior research, some comparisons are vague. The phrase “this study found increased differences in brain activation across PA levels” should be contextualized within previous findings. How do these differences compare quantitatively or qualitatively with past studies?
**Response:** Thanks for your suggestion. We have now revised the sentences as follows:

*“Our study revealed that brain activation differences across PA levels were more pronounced in older adults, who recruited broader neural resources during the switching task compared to younger adults. This expanded activation pattern in older adults suggests an age-related shift toward more distributed neural processing to maintain cognitive performance.”*

- Some cited studies (e.g., yoga practitioners vs. controls) focus on specific types of PA but are not directly linked to the present findings. A stronger rationale for including these comparisons is needed.

**Response:** Thanks for your suggestion. We have now revised the sentences as follows:

*“Importantly, the effects of PA on brain activation patterns appear to be modulated by activity type. For instance, while our study focused on general PA levels, specialized activities like yoga, which combine physical movement with mindfulness, have been shown to elicit distinct neural patterns. Gothe et al. (2018) reported reduced dorsolateral prefrontal cortex activation in yoga practitioners during working memory tasks, potentially reflecting greater neural efficiency through meditative practice. This complements our findings by demonstrating that how PA is implemented (e.g., aerobic exercise vs. mind-body practices) may determine its cognitive benefits. Similarly, another study found that PA's cognitive benefits are further influenced by lifestyle factors like sleep, with adequate sleep duration enhancing prefrontal activation during working memory tasks [52]. Together, these studies underscore the importance of considering both PA type and complementary lifestyle factors when interpreting neural outcomes.”*

4. The discussion acknowledges limitations, but some aspects require further elaboration. For instance, using self-reported PA data is mentioned as a limitation, but what potential biases might this introduce (e.g., recall bias, social desirability bias)?
- The suggestion for future research using the independent component algorithm is valid, but why is this method particularly useful for analyzing executive function and PA? A brief explanation of its advantages over Pearson’s correlation would be beneficial.

**Response:** Thanks for your suggestion to strengthen our limitations and future directions sections. We have elaborated on self-report biases and explained the independent component algorithm’s unique value for studying PA-brain networks. We have now revised the sentences as follows:

*“Additionally, PA was measured with a self-reported questionnaire. Participants may overestimate PA levels through recall inaccuracies [58]. Future studies should combine them with accelerometry or heart-rate monitoring for more precise dose-response characterization. Lastly, while Pearson’s correlations identified localized PA-related activation differences, future studies could apply independent component analysis to examine large-scale network dynamics. Independent component analysis decomposes fMRI data into functionally coherent networks (e.g., frontoparietal control network) without a priori seed selection [59]. This data-driven approach may better capture how PA modulates distributed neural circuits supporting executive functions, complementing our task-activated findings.”*

5. Some correlations are interpreted in a way that may suggest causality. For example, stating that PA “enhances” prefrontal lobe functioning should be revised to indicate association rather than direct causation due to the cross-sectional nature of the study.
**Response:** Thanks for your suggestion. We have replaced all causal language with more precise terms indicating association (e.g., "is associated with", "correlates with") throughout the discussion section.

- The study should acknowledge alternative explanations for the observed brain activation differences, such as genetic predispositions or pre-existing cognitive differences that might confound the results.

**Response:** Thanks for your suggestion. We have added the alternative explanations for the observed brain activation differences. The newly added sentences are as follows:

*“Firstly, the cross-sectional design limits more in-depth analyses of the correlation and changes in PA and executive function. The observed brain activation differences may reflect genetic predispositions or baseline cognitive differences rather than PA effects per se. For example, individuals with naturally more efficient neural processing may be both more physically active and show different activation patterns. Future studies can adopt longitudinal and RCT study designs, controlling for genetic and cognitive baseline differences would help clarify these relationships.”*

6. Some sentences are overly complex, reducing readability. For instance, “Exercise-induced neurocognitive changes affect task switching and working memory in older adults, especially with cognitive impairment” could be reworded for clarity.
- There are minor grammatical inconsistencies (e.g., "Besides, PA was measured..." → "Additionally, PA was measured..."). Consider revising for formal academic tone.

**Response:** Thanks for your suggestion. We have now revised the complex sentence and corrected grammatical inconsistencies as follows:

*“Physical exercise modifies neural function, particularly improving task-switching and working memory abilities in older adults—with pronounced effects in those experiencing cognitive decline.”*

*“Additionally, PA was measured with a self-reported questionnaire.”*

Conclusion
The conclusion provides a concise summary of the study’s results but could be expanded to reinforce the study's contributions. Instead of merely restating findings, briefly highlight their broader implications for cognitive aging and PA.
- The statement “inactive older adults needed to activate more brain regions to complete executive function tasks” should specify whether this supports a compensatory mechanism or reflects inefficiency in cognitive processing.
- The conclusion should discuss the relevance of the findings in a broader context. For example, what do these results suggest about PA as a potential intervention for cognitive decline?
- Consider adding a sentence on how these findings align with or challenge existing literature, reinforcing the study’s contribution to the field.
- The conclusion does not mention any limitations, which should be briefly acknowledged to provide a balanced interpretation. Reference issues like the cross-sectional design, self-reported PA measures, or sample size constraints.
- Suggesting potential future research directions, such as longitudinal studies or intervention trials, would strengthen the conclusion.
- The final paragraph is dense with technical terms (e.g., "right medial and paracentral cingulate gyrus (BA 6)"), which may make it less accessible. Consider summarizing key findings in simpler terms before mentioning specific brain regions.
- The conclusion could be more structured, moving from a summary of findings to broader implications and then future research directions.

**Response:** Thanks for your suggestion. We have now revised the sentences as follows:

*“Our study yields important insights into how physical activity (PA) relates to neural processing of executive functions across adulthood: (1) Physically inactive older adults showed more widespread brain activation during executive tasks, suggesting either compensatory recruitment or less efficient neural processing; (2) Active young adults demonstrated more focal activation patterns associated with better performance; and (3) Specific prefrontal regions (including the cingulate and dorsolateral frontal gyri) emerged as particularly sensitive to PA effects. These findings suggest that regular PA, particularly activities engaging prefrontal networks, may help maintain efficient brain function during aging. This reinforces PA's potential as a accessible, low-cost strategy to support cognitive vitality in older adults.”*

Reviewer #2: Title: fMRI insights into differential brain activation and physical activity in older adults
Author: Huiqi SONG, Jie FENG, Yingying WANG, Qichen ZHOU, Chenglin ZHOU, Jing JIN
Date of review: 02/21/2025

Strengths of the article
1. Relevance of the subject
The article deals with a subject of major interest in cognitive neuroscience and ageing, namely the impact of physical activity on executive function and brain activation. The research question is well motivated and supported by references.
2. Well-detailed methodology
The study uses well-established tasks to assess executive function (Flanker, N-back, Switching). The use of fMRI to analyse brain activation is relevant and justified.
3. Interesting results
The study shows interesting differences in brain activation depending on the level of physical activity. The finding that physically active adults require less activation to perform executive tasks is consistent with the hypothesis of better cognitive efficiency.
**Response:** Thanks for your comments.

Weaknesses and suggestions for improvement
1. General comments regarding the document
Q1. There are several spelling errors: the term executive functions is plural. It is also written “recreation” instead of “reaction” in several places in the document.
**Response**: We sincerely thank you for catching these typographical errors. We have carefully proofread the manuscript and corrected all spelling errors.

2. Title page
“fMRI insights into differential brain activation and physical activity in older adults”
Q2. Why don't you mention the term “executive functions” in the long title?
**Response**: Thanks for your suggestion. We agree that explicitly mentioning 'executive functions' would better reflect the study's focus. We have revised title to *“fMRI insights into differential brain activation, executive function, and physical activity in older adults”.*

3. Abstract
“Methods: This cross-sectional study involved 51 Han Chinese adults (22 young, 31 older) who were categorized as physically active (≥3000 MET-min/week) or inactive (<3000 METmin/ week).”
Q3. Please explicit the term “MET-min/week”.
**Response**: Thanks for your suggestion. We have explicit the term “MET-min/week. We have now revised this sentence as follows:

*“This cross-sectional study involved 51 Han Chinese adults (22 young, 31 older) who were categorized as physically active (≥3000 metabolic equivalent (MET)-min/week) or inactive (<3000 MET-min/week).”*
 
4. Methods
- Study design and participants
“The inclusion criteria were: (1) Han Chinese adults aged 18 years or older; (2) had more than 10 years of education”
Q4. What was the exact upper age limit in the young group? What were the exact lower and upper age limits in the older group?

**Response:** Thanks for your suggestion, the age range for young group was 19-27 and the age range for old group was 59-69. We have added the age range in Table 1.

Q5. The mean educational level in the older group is 11.10 +/- 1.97. It means that 1 or more participant(s) had less than 10 years of education. How do you explain that discrepancy?
“A total of 51 participants were included, including 22 young adults and 31 older adults.”

**Response:** We apologized for the typo in this number. The educational threshold should be 9 years, including 6 years for elementary school and 3 years for junior high school, based on China's compulsory education system structure. We have now revised this sentence as follows:

*“The inclusion criteria were: (1) Han Chinese adults aged 18 years or older; (2) had more than 9 years of education; (3) were at low risk of cognitive decline.”*

Q6. I do not understand this sentence as 22+31 = 53.

**Response:** We apologize for this error in reporting participant numbers. The correct total is indeed 53 participants (22 young + 31 older adults). We have now revised this sentence as follows:

*“A total of 53 participants were included, including 22 young adults and 31 older adults.”*

Q7. How was the sample size calculated? This information must be indicated according to the Strengthening the Reporting of Observational Studies in Epidemiology (STROBE) statement.
**Response:** Thanks for your suggestion. This study did not perform a formal a priori sample size calculation due to its exploratory observational design and the challenges in recruiting participants from a highly specific population, which necessitated snowball sampling—a common approach for hard-to-reach groups. In line with the STROBE statement (Item 10), we acknowledge that the sample size was determined by feasibility rather than statistical power, a recognized practice in hypothesis-generating fMRI studies where sample sizes are often constrained by practical considerations (e.g., cost, participant availability). We have stated this limitation in the discussion section. The newly added sentences are as follows:

*“Secondly, the relatively small sample size and lack of representativeness may limit the generalization of our findings to the population. The adoption of snowball sampling relies on existing participants to refer others, leading to a non-random sample. This can introduce selection bias, impacting the sample's representativeness of the population.”*

- Imaging data acquisition and analysis
Q8. Why did you not include imaging in the “Measurements” section?

**Response:** Thanks for your suggestion. The fMRI acquisition and analysis protocols were intentionally described in a separate ‘Imaging data acquisition and analysis’ subsection to avoid overcrowding the 'Measurements' section.

Q9. What corrections for multiple testing were applied? They must be reported in each table.

**Response:** Thanks for your suggestion. We have added the details in the Second-Level Analysis. We have now revised this sentence as follows:

*“The independent t-test was conducted for whole-brain activation within each group with 0 as the reference value. Multiple comparison correction for whole-brain data was applied using Gaussian random field (GRF) theory.”*

In Table 3, we have added the relevant descriptions, which are expressed in detail as follows:

*“Note: Activation of all listed voxels was statistically significant at a GRF corrected p < 0.01.”*

Q10. How did you select ROIs?
**Response:** Thanks for your valuable question. In this study, the ROI was defined based on the significant activation clusters identified through whole-brain analysis in three executive function tasks (flanking tasks, N-back tasks, and switching tasks). ROI is generated using spheres with a radius of 6mm, centered on the peak voxel coordinates within each activation cluster.

*“Regions of interest (ROIs) were generated with a 6 mm-radius sphere centered on the peak voxel coordinate within each activation cluster [39].”*

- Statistical methods
Q11. Statistical methods must be described (see the STROBE statement). There is no indication of which statistical tests were used to analyze data other than brain imaging data. Nor is it indicated which dispersion measure is presented in the article: standard deviation or standard error of the mean? I recommend to add a section 2.4. “Statistical methods” or “Statistical analyses”.
**Response:** Thanks for your suggestion. We have added the “statistical analyses” to the methods section. We have now revised the sentences as follows:

*“The continuous data variables were displayed as mean ± standard deviation (SD), while categorical data were presented as participant count and percentage. Following adjustment for sex and education level, a two-way ANOVA was performed to examine the impact of physical activity (PA) levels (low-to-moderate, high) and age groups (young adults, older adults) on cognitive function. A Bonferroni post-hoc test was conducted to assess differences. All statistical analyses were carried out using SPSS version 27.0, with a significance level set at 0.05.”*

5. Results
“Among 52 participants who agreed to participate in this study”
Q12. Why were 51 participants included and 52 participants who agreed to participate?

**Response:** We apologize for this error in reporting participant numbers. We have now revised the sentences as follows:

*“Of the 53 enrolled participants, 41 were included in the data analysis, while 12 were excluded due to scan absence (n=5), withdrawal (n=5), or incomplete questionnaires (n=2). Table 1 displays participant characteristics. The young adult group (n=21; 23.0 ± 2.12 years, 42.9% female) comprised 52.4% physically active individuals, compared to 60.0% in the older adult group (n=20; 63.30 ± 2.36 years, 45.0% female). Excluded participants were significantly older than included participants (64.3 ± 2.1 vs. 42.6 ± 20.5 years, p < 0.001), with no other demographic differences.”*

Q13. What was the mean physical activity level in each group? This must be indicated in the Table 1.

**Response:** Thanks for your suggestion. We have indicated the mean physical activity level in each group in Table 1.

Q14. How did you choose the physical activity cut-off between physically active and physically inactive (3000 MET-min/week)? Is it relevant to choose to use the same cut-off for young and older groups? One may argue that an older person will be considered as active with less activity than a younger person.

**Response:** Thanks for your suggestion. The selection of the 3000 MET-min/week threshold was based on established ACSM guidelines and previous research. While it is a valid point that older individuals may achieve the same health benefits with lower levels of physical activity compared to younger individuals, the consistency in cut-off across age groups allows for a standardized comparison within our study sample. We have now revised this sentence as follows:

*“Participants were classified into the physically active group with PA achieving ≥3000 MET-min/week and the physically inactive group with PA achieving <3000 MET-min/week [26].”*

**Reference**

26. Andersen E, Kjellså I, Hjellset VT, Henjum S. Insufficient physical activity level among Sahrawi adults living in a protracted refugee setting. BMC Public Health. 2021;21(1):166. doi: 10.1186/s12889-021-10217-w.

Q15. Results must indicate the statistics value (for example: t value if a Student t test has been used or the F value if an ANOVA has been performed) wherever necessary. P values are not sufficient.
**Response:** Thanks for your suggestion. We have added the mean differences (95% CI) and effect sizes in Table 2 to help readers understand the magnitude of observed differences.
 
6. Table 1.
“Data are presented as mean (SD) or number (percentage).”
Q16. I do not understand this sentence. It seems that some lines indicate means +/- SD and that other lines indicate the number of participants with percentages in brackets. Is it correct?

**Response:** We thank the reviewer for identifying this ambiguity in our data reporting description. We have now revised the sentences as follows:

*“Data are presented as mean ± SD or number (percentage).”*

Reviewer #3: The study is well-structured and explores an important topic. However, minor revisions are needed to clarify the justification for the 3000 MET-min/week threshold and ensure greater scientific accuracy and clarity.

**Response:** Thanks for your suggestion. We have clarified the justification for the 3000 MET-min/week threshold. We have now revised this sentence as follows:

*“Participants were classified into the physically active group with PA achieving ≥3000 MET-min/week and the physically inactive group with PA achieving <3000 MET-min/week [26]. The IPAQ-SF has good reliability and validity in Chinese adults [27].”*

**Reference**

26. Andersen E, Kjellså I, Hjellset VT, Henjum S. Insufficient physical activity level among Sahrawi adults living in a protracted refugee setting. BMC Public Health. 2021;21(1):166. doi: 10.1186/s12889-021-10217-w.

27. Macfarlane DJ, Lee CCY, Ho EYK, Chan KL, Chan DTS. Reliability and validity of the Chinese version of IPAQ (short, last 7 days). Journal of Science and Medicine in Sport. 2007;10(1):45-51. doi: <https://doi.org/10.1016/j.jsams.2006.05.003>.
