# Supplementary material for: fMRI insights into differential brain activation, executive function, and physical activity in older adults
Source: PLoS One. 2025 Jun 25;20(6):e0327163. doi: 10.1371/journal.pone.0327163 (PMC12194046; doi:10.1371/journal.pone.0327163)
Supplement: Attachment — Submitted filename: Response_to_reviewers_auresp_2.docx [file pone.0327163.s006.docx]

**Response to reviewers**

Review Comments to the Author

The authors have provided references to support their use of the 3000 MET-min/week threshold (Andersen et al., 2021; Macfarlane et al., 2007). While these sources confirm the general applicability of the IPAQ-SF and its validity in certain populations, they do not sufficiently explain why this particular cutoff was chosen in the context of cognitive health or brain activation.

This threshold is not commonly used in neuroimaging or cognitive neuroscience research. Therefore, its selection in this study should be either:

- clearly justified with reference to prior work or guidelines related to brain health (e.g., WHO, ACSM), or

- explicitly presented as a novel, hypothesis-driven approach to examine possible neurocognitive associations.

Providing this clarification will strengthen the conceptual alignment between the study’s aims and its methodological framework.

**Response:** Thanks for your valuable suggestions. We have added justification of the 3000 MET-min/week threshold. We have now revised this sentence as follows:

*“Participants were classified into the physically active group with PA achieving ≥3000 MET-min/week and the physically inactive group with PA achieving <3000 MET-min/week. This threshold aligns with the high activity category defined in the IPAQ scoring protocol [24]. The higher threshold is selected based on evidence that found PA was positively associated with brain health in older adults [26].”*

**Reference**

24. Craig CL, Marshall AL, Sjöström M, Bauman AE, Booth ML, Ainsworth BE, et al. International physical activity questionnaire: 12-country reliability and validity.

Medicine & science in sports & exercise. 2003;35(8):1381-95.

26. La Hood A, Moran C, Than S, Lu A, Collyer TA, Beare R, et al. Associations between physical activity and brain structure in a large community cohort. Scientific Reports. 2025;15(1):18896. doi: 10.1038/s41598-025-04010-7.
